# Editorial: The effects of circulating non-sex hormones in cardiovascular disease

**DOI:** 10.3389/fendo.2022.1065155

**Published:** 2022-12-20

**Authors:** Su-Kang Shan, Tong Yan, Ling-Qing Yuan

**Affiliations:** ^1^ National Clinical Research Center for Metabolic Diseases, Department of Metabolism and Endocrinology, The Second Xiangya Hospital, Central South University, Changsha, China; ^2^ Center for Obesity and Metabolic Health, Department of General Surgery, The Third People’s Hospital of Chengdu and the Affiliated Hospital of Southwest Jiaotong University, Chengdu, Sichuan, China; ^3^ Center for Diabetes Care, Pangang Group Chengdu Hospital, Chengdu, Sichuan, China

**Keywords:** non-sex hormones, cardiovascular disease, irisin, humanin, SFRPs, FT4/FT3 ratio, vitamin D, α-klotho

Hormones are chemical messengers that transmit signals between cells and organs. They are transmitted through blood or tissue fluid, acting on corresponding cells and tissues, and are responsible for coordinating the activities of different parts of the body. Hormones can act on adjacent tissues and even the whole body through endocrine, paracrine, and autocrine manners. Normal levels of hormones play an important role in the stability of the cardiovascular system. For example, the activation of the renin-angiotensin-aldosterone system (RAAS) can enhance cardiac contractility, constrict peripheral blood vessels, regulate water and sodium retention, and then participate in pathophysiological cardiovascular disease (1, 2). In addition to the classic RAAS, sex hormone levels are associated with the risk of cardiovascular disease. Higher androgen and lower estrogen levels were reported to be associated with cardiovascular disease risk factors in women (3).

A hormone has multiple functions, meanwhile, multiple hormones can participate in the regulation of physiological functions through synergy or antagonism. Researchers have gradually discovered that hormones secreted by tissues/cells other than endocrine glands are actively involved in the regulation of the cardiovascular system. In this topic, the authors mainly discussed the effects of non-sex hormones on the cardiovascular system, which would help deepen the understanding of circulating hormones on cardiovascular diseases and provide ideas for the development of potential diagnostic and therapeutic targets for cardiovascular disease.

The topic received six papers, including three reviews and three articles.

Irisin is a myokine secreted mainly by cardiomyocytes and skeletal muscle cells, and the classic functions of irisin are browning white adipose tissue and generating heat. Yang et al. introduced myokine irisin and summarized the controversial role of irisin in the clinical management of coronary heart disease. The identification of irisin levels measured by different methods led to the inconsistency of cross-sectional studies, making it hard for the clinical application of irisin. Moreover, the authors appealed for a more in-depth and repeatable exploration of its pathophysiological role in coronary heart disease to be conducted.

Cai et al. focused on a new relationship between aging-related cardiovascular diseases (ACVD) and humanin (HN), an endogenous peptide encoded by mitochondrial DNA. HN protected cardiomyocytes, endothelial cells, and fibroblasts from oxidative stress, highlighting its protective role in atherosclerosis, ischemia-reperfusion injury, and heart failure. Furthermore, the author summarized the signaling pathways associated with the HN effects on redox signals. And they proposed that HN may be a candidate drug for ACVD.

Secreted frizzled related proteins (SFRP) family members are antagonistic inhibitors of the Wnt signaling pathway and are involved in regulating metabolic and cardiovascular disease. Guan et al. reviewed current knowledge about SFRPs and their role in adipocyte differentiation, obesity, diabetes, insulin resistance, angiogenesis, myocardial injury, and coronary artery disease. SFRP1-5 have been identified and named. The functions and involved pathways of SFRP family members are demonstrated by various SFRP knockout mice, as pointed out by the authors.

A prospective cross-sectional study conducted by Zhang et al. recruited ischemia and no obstructive coronary artery disease patients (INOCA), which were divided into coronary microvascular dysfunction (CMD) group and none coronary microvascular dysfunction group by using myocardial perfusion imaging (MPI) and dynamic single-photon emission computed tomography (D-SPECT). Their result showed that an increased free triiodothyronine/free thyroxin(FT3/FT4) ratio was relatively correlated with an increased risk of CMD, FT4/FT3 ratio was expected to provide a novel biomarker for early prevention and risk stratification for CMD in INOCA patients.

A series of prospective studies showed that low concentrations of vitamin D were correlated with relatively higher risk and severity of the cardiovascular disease. However, no evidence of patients with type 1 diabetes (T1D) eligible for simultaneous pancreas- transplantation has been found yet. Buksińska-Lisik et al. conduct a prospective cross-sectional study in T1D eligible for simultaneous pancreas-kidney transplantation or pancreas transplant alone. They found a high prevalence of coronary artery disease in T1D patients eligible for pancreas transplantation. Vitamin D deficiency may serve as an extra cardiovascular risk factor among pancreas transplant candidates with T1D. Indicating the rationality of measurement and supplementation of 25(OH)D concentration in type 1 diabetic patients eligible for pancreas transplantation.

α-Klotho was an aging-related gene whose protein could act as a circulating hormone (4). Recently, scientists have focused on the protective role of circulating α-Klotho in the risk of cardiovascular diseases (CVD) (5–7). However, the randomized controlled trial is unavoidably biased by reverse causation and confounding factors. Sun et al. conduct the Mendelian randomization study (MR), utilizing genetic variants as instrumental variables to explore the associations between α-Klotho and cardiovascular diseases (outcomes). As genetic variants were stable, the Mendelian randomization study avoid reverse causation in observational studies (8). Finally, evidence was found for a protective effect of circulating α-Klotho on the prevention of atrial fibrillation risk. However, no significant causal association between genetically predicted circulating α-Klotho levels and risk of CAD, hear failure, stroke, ischemic stroke (IS), or IS subtypes was found.

Taken together, the Research Topic summarizes the current knowledge and focuses on new insights into the effects of circulating non-sex hormones in cardiovascular disease. However, the complete molecular mechanism and its underlying role still warrant further investigation.

## Author contributions

All authors listed have made a substantial, direct, and intellectual contribution to the work and approved it for publication.
